# Clinical Effects of Mindfulness-Based Intervention in Patients With First Episode Psychosis and in Individuals With Ultra-High Risk for Transition to Psychosis: A Review

**DOI:** 10.3389/fpsyt.2019.00797

**Published:** 2019-10-31

**Authors:** Philippe Vignaud, Karen T. Reilly, Clément Donde, Frédéric Haesebaert, Jérôme Brunelin

**Affiliations:** ^1^INSERM U1028, CNRS UMR5292, PSYR2 Team, Lyon Neuroscience Research Center, Université Claude Bernard Lyon 1, Lyon, France; ^2^Centre Hospitalier Le Vinatier, Bron, France; ^3^INSERM U1028, CNRS UMR5292, ImpAct Team, Lyon, Neuroscience Research Center, Université Claude Bernard Lyon 1, Lyon, France

**Keywords:** mindfulness, MBCT, MBSR, ultra-high risk for transition to psychosis, first psychotic episode

## Abstract

**Objectives:** Recent clinical studies and meta-analyses have reported the clinical effects of mindfulness-based interventions as a complementary treatment for patients with schizophrenia, but their possible efficacy in patients with first episode of psychosis (FEP) and in individuals with ultra-high risk (UHR) of transition to psychosis is less clear. Here, we investigated the current evidence on the usefulness of mindfulness-based interventions in these two populations.

**Methods:** We conducted a systematic search of the literature according to the PRISMA guidelines.

**Results:** Among the 102 references retrieved, 9 responded to the inclusion criteria (8 in FEP patients and 1 in UHR individuals). In FEP patients, mindfulness interventions are well-tolerated and have a satisfactory level of adherence. The clinical benefits consist primarily of reduced anxiety and sadness and improved quality of life. None of the studies reported any increase in positive symptoms.

**Conclusion:** Future sham-controlled studies with large sample sizes are needed to definitively conclude on the clinical interest of mindfulness-based interventions in FEP patients and UHR individuals as well as to understand their underlying mechanisms of action.

## Introduction

Schizophrenia is often preceded by a prodromal phase which can sometimes last for several years. It has been suggested that an early intervention during this prodromal phase could decrease the burden associated with the disorder, increase quality of life and global functioning, and delay transition to psychosis (1, 2). The existence of this prodromal phase, and the search for an early intervention, has seen the emergence of the concept of ultra-high risk (UHR) for transition to psychosis. In people aged between 14 and 30 years old, the UHR population can be divided into three clinical presentations. First, those with a schizotypal personality disorder or a first-degree relative with a psychotic disorder, as well as a 30% reduction in overall functioning level on the Social and Occupational Functioning Assessment Scale (SOFAS). Second, those with attenuated (either in intensity or frequency) psychotic symptoms (APS). Third, those with brief, limited, intermittent, and psychotic symptoms (BLIPS) in which symptoms persist for less than a week and disappear spontaneously (3). In each of these three clinical presentations symptoms are present in the year before and have progressed for fewer than 5 years (4). The Comprehensive Assessment of At Risk Mental State (CAARMS) is the most widely accepted psychometric scale for evaluating these at-risk states, and psychotic transition is identified by the occurrence of a first episode of psychosis (FEP). Early interventions classically focus on UHR individuals and FEP patients 2–5 years after their FEP (5, 6), and are based on a clinical staging model of the disorder (7). Although these interventions do not substantially reduce the number of people who transition to psychosis, they can delay disease onset (8; 9). They also reduce the duration of untreated psychosis (10). This last point is important, as a long period of untreated psychosis is not only associated with more severe symptoms, but also with greater alteration in social functioning, less efficient global functioning, and impaired quality of life (11). In addition to reducing the duration of untreated psychosis, these interventions are also interesting because they aim to reinforce the patient’s sense of competence and their experience of self-efficacy, and to promote stress management (12, 13), all of which are skills that fall under the umbrella term self-empowerment.

Patients with schizophrenia have major functional deficits (14, 15) and the disorder is associated with significant health costs (16). Although UHR or FEP patients do not systematically develop schizophrenia (17), they can be affected by other psychiatric disorders and they increasingly seek help and demand care. As a consequence, there is growing interest in this population, a fact reflected by the current state of the scientific literature.

Mindfulness meditation is a practice that aims to achieve a state of consciousness in which the person intentionally pays attention, in the present moment, without judgment, to the unfolding experience moment by moment (18). Historically, this practice stems from the Buddhist religion, and it was first adapted to a therapeutic context (mindfulness-based intervention) in the form of a group intervention to improve the management of stress and chronic pain: the Mindfulness Based Stress Reduction (MBSR) program. Inspired by the MBSR program, and enriched with elements from cognitive and behavioral therapy (CBT), the Mindfulness Based Cognitive Therapy (MBCT) program was subsequently developed to treat depression, and above all, prevent depressive relapse (19).

While typical indications for mindfulness-based interventions include depression treatment and relapse prevention, stress management, chronic pain, and addiction (20), in recent years there has been increasing interest in applying this technique to schizophrenia spectrum disorders (21). Khoury and colleagues (22) performed a meta-analysis of clinical trials of mindfulness-based interventions in schizophrenia and found a significant overall therapeutic effect, low to moderate (for pre- versus post- intervention analysis, d = 0.52, p < 0.0001, for the active versus control group analysis, d = 0.47, p < 0.0001). Specifically, the therapeutic effect was associated with reduced negative symptoms (d = 0.75, p < 0.01), lower depression and anxiety (d = 0.43, p < 0.0001), fewer hospitalizations (d = 0.60, p < 0.0001), and improved quality of life (d = 0.49, p < 0.05). There was also a non-significant trend towards a reduction in positive symptoms (p = 0.081).

Even though attenuated psychotic symptoms appear to best predict transition to psychosis, UHR individuals frequently present with other non-specific symptoms, including depression and anxiety. Psychotherapy interventions, in particular of the CBT type, appear to reduce transition to psychosis (23). While the medical management of FEP patients also includes pharmacotherapy, principally with antipsychotics, their use in UHR individuals is problematic, given the questionable effectiveness of antipsychotics in this population and the fact that many individuals will not transition to a psychotic disorder (13, 24).

FEP patients have a symptomatology close to that of patients with schizophrenia spectrum disorder, in whom the therapeutic effect of mindfulness-based interventions has been demonstrated. The issues of stress management and treatment of post-critical anxiety and depression are also predominant in FEP patients. Given this context, we were interested in investigating the clinical potential of mindfulness in the management of FEP patients and UHR individuals. The aim of this review was to perform a systematic inventory of clinical studies assessing mindfulness in FEP patients and UHR individuals, to describe them, and to look at whether the interventions are potentially useful.

## Method

A literature review was performed following the PRISMA guidelines (25) with the aim of describing clinical studies assessing mindfulness in FEP patients and UHR individuals.

### Inclusion Criteria of Studies

Articles were included based upon the following criteria: i) written in English and published in a peer-reviewed journal, ii) study participants were either FEP patients or UHR individuals (as defined in the Introduction by 4), iii) a mindfulness-based intervention was proposed to participants, and iv) a clinical evaluation was performed before and after the mindfulness-based intervention. Exclusion criteria were: i) did not contain a mindfulness-based intervention, ii) study participants suffered from chronic schizophrenia with more than 5 years of disease progression, and iii) case studies.

### Search Strategy

We performed a systematic article search using five databases: PubMed, Cochrane, PsycINFO, PubPsych, and psycEXTRA from the beginning of online records until the 1^st^ of April 2019 using the key words: (mindfulness) AND (brief limited intermittent psychotic symptoms OR attenuated positive symptoms OR at risk of psychosis state OR early psychosis OR first psychotic episode OR early schizophrenia OR attenuated psychotic symptoms OR early psychotic experiences OR early intervention in psychosis OR first episode of psychosis). Two authors (PV, JB) performed this search independently, and any disagreements concerning inclusion/exclusion of an article were resolved after discussion with a third author (FH). We also used the “similar articles” function in PubMed to search for other articles as well as the bibliographies of articles included based upon the above-mentioned criteria.

### Data Extraction

When available, we extracted the following data from each article: sample size, type of participants (UHR/FEP), study design, participant age and gender, inclusion and type of control group, characteristics of the mindfulness-based intervention (type of intervention, number of sessions), clinical evaluation time points, and judgement criteria.

## Results

**Figure 1:**
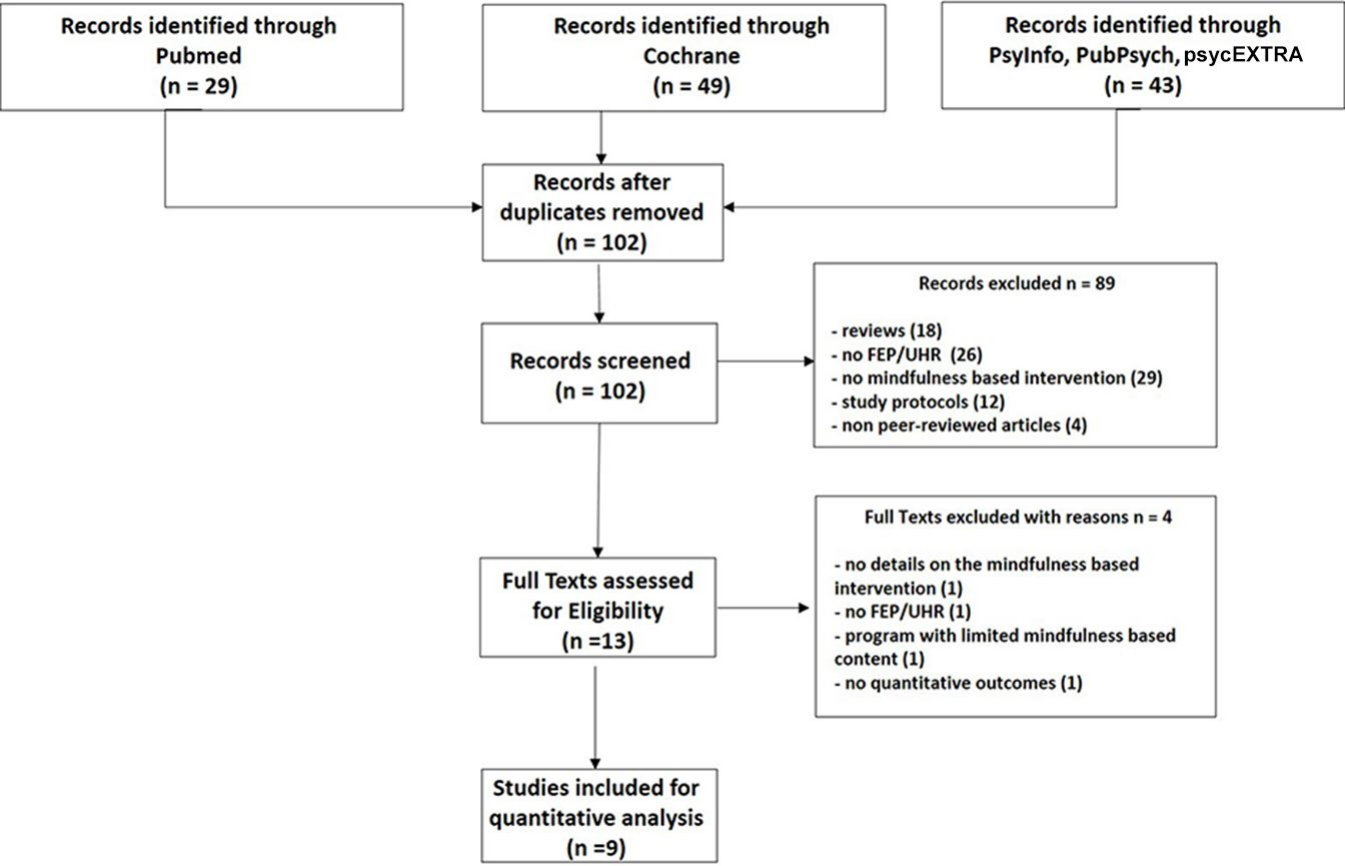
PRISMA (Preferred Reporting Items for Systematic Reviews and Meta-Analyses) flow diagram.

As shown in **Figure 1**, 102 articles were identified after exclusion of duplicates, of which 13 full-text articles were read. The main reasons for exclusion were: literature review, ongoing study, participants not UHR or FEP, and absence of mindfulness-based intervention. Among the 13 full-text articles read, 4 articles were excluded. The first excluded study was a pilot study evaluating the effect of a multimodal smartphone application (psychoeducation, cognitive remediation, peer discussion forum) of which the mindfulness-based intervention represented only a small part that could not be evaluated without substantial bias (26). One study did not provide any details about the mindfulness-based intervention, and one study included patients with chronic schizophrenia. The last excluded article was an open study including nine FEP participants and only qualitative data were available (27). Thus, nine articles were retained for inclusion in the review, of which one included UHR individuals while eight included FEP patients.

In 2012, Moorhead and colleagues conducted an open study with 19 participants (11 patients from an early intervention unit; 5 healthcare staff members; 3 caregivers) to evaluate the feasibility of a mindfulness-based intervention with outpatients in an early intervention service. The originality of this study was that participants in the program were patients receiving treatment in the service, as well as their caregivers and healthcare professionals. The 8-week program consisted of weekly 1-h sessions, and on average 11.25 (out of 19) people participated in each session. The program was viewed as both acceptable and as having a positive effect on everyday life. A global score evaluating distress level was created for the study. When all participants were analyzed together this score decreased significantly after the program (Idiosyncratic measure *before* = 300; Idiosyncratic measure *after* = 90; Mann–Whitney *U* test *z* score = −3.82, *p <* 0.01). The patient sub-group exhibited a clinical benefit of the program but the decrease in their global distress score was not significant (Idiosyncratic measure *before* = 272.5; Idiosyncratic measure *after* = 205; Mann-Whitney U test *z score* = −1.76, *p* = 0.079).

The Van der Valk team (28) studied the feasibility of a mindfulness-based intervention with FEP patients. In a non-controlled study, they included 19 FEP patients, 18 of whom completed the whole program and reported being highly satisfied. One patient reported feeling intense distress and was unable to complete the program. The authors interpreted this, *a posteriori*, as related to the patient’s misunderstanding of the instructions for practicing mindfulness. The authors administered the Symptoms Check-List 90 before and after the program and found a significant reduction in agoraphobia and psychoneuroticism (SCL; psychoneuroticism effect size d = 0.44; agoraphobia d = 0.43) as well as a trend towards a reduction in negative symptoms and disorganization.

In a non-controlled study, Samson and Mallindine (29) evaluated the feasibility of a mindfulness-based intervention group in a group of patients admitted to an early intervention unit. Eight FEP patients participated in an 8-week program which consisted of weekly 90-min sessions. The participation rate was 57.81%. They administered three scales before and after the program: the Clinical Outcomes in Routine Evaluation – Outcome Measure (CORE – OM), the Southampton Mindfulness Questionnaire (SMQ) and the Depression Anxiety Stress Scale – Short Form (DASS – 21). The CORE-OM score decreased after the program (average *before* = 1.25; average *after* = 0.88, t7 = 1.41, p = 0.20), as did the DASS score (average *before* = 31.63; average *after* = 14.38, t7 = 1.9, p = 0.10), while the SMQ score increased (average *before* = 44.75; average *after* = 58.75, t7 = -1.86, p = 0.11). None of these changes were statistically significant.

Another study conducted by Khoury and collaborators (30) in FEP patients evaluated the feasibility, acceptability, and the potential clinical relevance of an intervention based upon the practice of mindfulness (the *Compassion, Acceptance and Mindfulness* program). Twelve patients were included in this non-controlled study in which the psychotherapeutic intervention was comprised partly of concepts borrowed from mindfulness and partly of concepts borrowed from Acceptance and Commitment Therapy (ACT, a third-wave CBT). The intervention was evaluated using the Brief Psychiatric Symptoms Rating Scale (BRPS) and the Cognitive Emotion Regulation Questionnaire (CERQ) and patient insight was measured using the Beck Cognitive Insight Scale (BCIS). Very few patients dropped out of the study, and those who remained reported being satisfied with the intervention. Management of negative emotions improved significantly after the intervention [negative emotion sub-score on the CERQ: average before = 53.17 (SD 9.21); average 3 months after the final session = 61.90 (SD 8.08); p = 0.007; d = 1.003], and depression and sadness scores tended to improve [depression anxiety score on the BPRS: average before = 10.50 (5.20); average 3 months after the final session = 8.10 (2.81); p = 0.082, d = 0.676]. In contrast, the authors observed no significant changes in positive symptoms, insight, or global functioning (Social Functioning Scale).

In 2016, Tong and colleagues conducted a study with a mixed methodology including quantitative and qualitative outcomes. These authors focused on the clinical effect of the mindfulness-based intervention. They included 14 patients with less than 5 years of disease progression. The mindfulness-based intervention program was largely inspired by the group-based Mindfulness-Based Stress Reduction (MBSR) program. The originality of their program was that several of the sessions were conducted outside the center, with the idea of facilitating generalization of the practice to everyday life situations. Their quantitative measures showed improvements in anxiety and depression scores: Beck Depression Inventory – II: 14 +/− 12.34 (before); 5.64 +/− 2.26 (after), and quality of life (Short Form - 12: 61.15 +/− 23.67 (before); 86.56 +/− 6.43 (after)), but no change in scores measuring positive symptoms.

The study by Wang and collaborators (31), which is good methodologically, is a multi-center, randomized, and controlled study that included a total of 138 patients. In this study they evaluated the effect of an intervention that included a mindfulness-based intervention (MBCT) in combination with psychoeducation, and compared it with a control group that received psychoeducation only and another group that received standard care only. Despite the length of the intervention (24 weeks, 12 2-h sessions once a fortnight), very few participants dropped out. The mindfulness + psychoeducation group showed a statistically significant improvement in quality of life [Specific Level of Functioning Scale (SLOF): mindfulness + psychoeducation: (before) 145.2 ± 16.0; (6 months after final session) 175.8 ± 13.0; psychoeducation only: (before) 142.9 ± 15.0; (6 months after final session) 153.5 ± 12.1; standard care only (before) 149.8 ± 16.5; (6 months later) 136.0 ± 18.0], fewer hospitalizations, and an improvement in both positive symptoms [positive symptom sub-scale of the PANSS: mindfulness + psychoeducation: (before) 26.9 ± 8.3; (6 months after final session) 16.9 ± 6.0; psychoeducation only: (before) 26.3 ± 7.0; (6 months after final session) 23.0 ± 9.1; standard care only (before) 26.5 ± 7.0; (6 months after final session) 28.9 ± 9.5) and negative symptoms (negative symptom sub-scale of the PANSS: mindfulness + psychoeducation: (before) 18.8 ± 7.4; (6 months after final session) 14.8 ± 5.2; psychoeducation only: (before) 18.0 ± 7.3; (6 months after final session) 18.8 ± 8.9; standard care only (before) 18.1 ± 8.1; (6 months after final session) 20.8 ± 9.8]. These improvements were significant compared with both the psychoeducation only and the standard care only groups. While these results are clearly encouraging, the authors caution that their patients had a high sociocultural level and good family support, factors that likely contributed to program adhesion and to its beneficial outcomes.

In a controlled study of an intervention versus standard care, MacDougall and colleagues (32) evaluated the acceptability and feasibility of the Mindfulness Ambassador Program (MAP; the focus of this program is on reinforcement of interpersonal relationships and social emotional learning) in a sample of 21 FEP patients. According to scores on the Client Satisfaction Questionnaire, participants were highly satisfied (CSQ = 30.2; SD = 1.6). Among the 21 patients recruited (87.5% of eligible patients accepted to participate in the study) 2 from each group dropped-out for reasons unrelated to the study content. The Profile of Moods States (POMS) questionnaire revealed a reduction in symptoms of both asthenia and depression [POMS-fatigue scale: MAP 3.7 (SD = 2.35), standard treatment 9.8 (SD = 3.77); POMS-depression scale: MAP 3.4 (SD = 3.64), standard treatment 12.5 (SD = 8.38)].

Another controlled study of an intervention versus standard care included 33 FEP patients and evaluated the feasibility of a multidisciplinary program including light physical activity, cooking classes with a focus on nutritional education, and mindfulness sessions (33). The mindfulness-based intervention program consisted of six 4-h group sessions. The program was viewed as highly feasible, as 88% of patients in the intervention group attended at least four of the six sessions. Furthermore, these patients had a significant decrease in their positive symptoms (t = −3.29; p = 0.002). However, no significant differences in cardio-metabolic measurements were observed, with the exception of a tendency towards a decrease in BMI in the active group (t = 1.77, p = 0.086).

The only study that examined the effect of a mindfulness-based intervention program in UHR individuals was a pilot study conducted by Alvarez-Jimenez and colleagues (34). They used the CAARMS criteria to recruit 14 UHR participants from an early intervention service. For 2 months the authors tested the effect of an internet-accessible application that contained several tools including: mindfulness learning modules; mindfulness exercises and behavioural activation; and a clinician-moderated online forum. The investigators’ *a priori* hypothesis was that the program would help users develop a number of different skills including: the feeling of empowerment, strengthening of self-efficacy, and building social and problem-solving skills. The primary objective of the study was to evaluate the feasibility and acceptability of the program. Seventy-two percent of participants connected to the application at least seven times and all participants contributed to the discussion forum, 57% of participants posted at least 28 messages on the forum. No clinically undesirable events were noted and the Global Functioning Social score revealed a significant improvement in social function (d = 1.83; p < 0,001). Personal skills (d = 0.70; p = 0.03) and mindfulness skills (d = 0.66; p = 0.04) also improved significantly, and there was a positive correlation between mindfulness skills and the feeling of well-being (r = 0.69; p = 0.01).

These results are summarized in the **Table 1**.

**Table 1:** Main results from included studies investigating group-based mindfulness-based interventions in first psychotic episode and at risk for psychosis populations.

Authors	No. Patients/type	Design	Average Age(SD)	Sex	N control group	Group-based Mindfulness Intervention	Timing of evaluations	Main results
35	19	open	ND	ND	NA	8 sessions, 1h, 1 session/week	post	- high participation and acceptability-Significant decrease in CORE-OM distress score (from 300 to 90; p < 0,01)
36	19/FEP	open	31.8(5.2)	14 M/5 F	NA	8 sessions, 1 h, 2 sessions/week for 4 weeks-formal practice	ND	-No increase in PANSS positive symptoms scores–Significant reduction in agoraphobia and psychoneurotic scores (SCL)-Tendency towards an improvement in non-aversion trait on the SMQ (p = 0.079)
29	10/FEP	open	29	7 M/3 F	NA	8 sessions, 1.5 h,1 session/week	ND	-Improvement in quality of life, sadness and anxiety scores (CORE-OM)-improvement on the SMQ-Non-significant clinical effect on all 3 scales
30	12/FEP	open	29,1(8,1)	8M/4 F	NA	8 sessions, between 60 and 75 min1 session/week	post&+3 months	-Significant improvement notably control of negative emotions (CERQ)-Significant reduction of depression and anxiety symptoms (BPRS).
37	14/< 5 years	open	47.0(12.2)	4 M/10 F	NA	7 sessions, 1.5h, 1 session/week	post	-Significant improvement in depression scores (DASS 21 and BDI II)-Improvement in general psychopathology (PANSS G2)-Significant improvement in quality of life (SF12)
31	138/< 5 years	RCT	23.8(6.8)	24 M/22 F	1 psycho-education group (n = 44)1 TAU group (n = 43)	12 sessions, 3 h, fortnightly,	+1 week&+6 weeks	-Significant improvement in global functioning, insight, and adhesion to Mindfulness program (SLOF)-Reduction of both positive and negative symptoms (PANSS)
32	17/FEP and < 3 years n	RCT	23.7	13 M/4 F	1 TAU group (n = 10)	12 sessions, 1 h, 1 session/week	post	- high level of satisfaction (CSQ score = 30.2 (1.6))- significant improvement in depression and asthenia sub-scores of the POMS
33	33/FEP and < 2 years	CT	19.5(3.8)	18 M/15 F	1 TAU group(n = 16)	6 sessions, 4 h,1session/week	+6 weeks&+12 weeks	–highly feasible- Reduction of positive symptoms at +12 weeks (QSAPS)
34	14/UHR	open	20.3(3.4)	3 M/11 F	NA	- web-based application	+2 months	-72% of participants connected at least 7 times and all participants participated in forum discussions- improvement in personal skills (d = 0.70; p = 0.03) and mindfulness skills (d = 0.66; p = 0.04)

SCL, the Symptoms C`heck-List 90; PANSS, Positive and Negative Symptoms Scale; SMQ, Southampton Mindfulness Questionnaire; BPRS, Brief Psychiatric Rating Scale; CORE-OM, Clinical Outcome in Routine Evaluation – Outcome Measure; DASS 21, Depression and Anxiety Symptoms Scale; BDI-II, Beck Depression Inventory; SLOF, Specific Level Of Functioning Scale; CSQ, Client Satisfaction Questionnaire; POMS, Profile of Mood States; -QSAPS, Quick Scale for the Assessment of Positive Symptoms; SF-12, Short Form 12 items. MADRS, Montgomery Asberg Depression Rating Scale. SD: Standard deviation; NA, Not applicable; ND, Not Done; TAU, Treatment as usual.

## Discussion

### Summary of Main Results

The currently available data show that mindfulness-based interventions are well-accepted and well-adhered to by FEP patients or patients in their first 5 years after a diagnosis of schizophrenia, and that they can have a clinical effect on symptoms of depression and anxiety. One large-sample study evaluating an intervention that combined mindfulness + psychoeducation also reported an improvement in both positive and negative symptoms in FEP patients (31). Several studies reported an improvement in quality of life and an effect on functioning. It is important to note that the majority of studies included FEP patients, while only one study evaluated the effect of a mindfulness program on UHR individuals. Group-based interventions were proposed in all included studies.

While the qualitative study by Ashcroft and collaborators (27) did not meet our inclusion criteria we think it worthy of mention here. This study evaluated the feasibility of a mindfulness-based intervention program in FEP patients, with a particular interest in how the patients experienced the practice. This non-controlled study included nine patients attending a center dedicated to FEP patients. Patients participated in a weekly 60-min group session and received a 10 min audio recording to encourage home practice. Semi-structured interviews were conducted 20 weeks after the first mindfulness session, at which time the nine included patients has participated in between 6 and 16 sessions. A person not involved in the group sessions subsequently analyzed the content of the filmed interviews and identified four main themes: « using mindfulness »; « making sense of mindfulness and coping »; « relating to people differently »; « understanding and accepting myself ». Overall, all patients were able to put in place a regular mindfulness practice and acquire skills classically targeted by mindfulness teachings. Using a similar approach of thematic analysis of formal interviews, the qualitative part of Tong and colleagues’ (37) study revealed 4 other themes: « Definition of the concept of mindfulness »; « rogram content »; « personal relevance of the program »; « understanding of symptoms and illness ». It is noteworthy that only a small number of patients mentioned experiencing difficulties practicing outside of the group context (5/14), or feeling pressured to maintain the practice in-between the formal group sessions (4/14). Taken together, these studies suggest that FEP patients experience mindfulness-based intervention programs as feasible.

### Acceptability and Effect on Sadness and Anxiety

The feasibility and acceptability of mindfulness-based interventions in patients with schizophrenia or schizophrenia-related disorders has long been a sensitive issue. Indeed, while mindfulness seeks to anchor the person in the present moment, it frequently also requires a calm and silent attitude that can intensify distressing experiences. Furthermore, the idea of mindfulness often evokes concern about the possibility of entering into altered states of consciousness (38). This concern has been fuelled by several case reports documenting a psychotic relapse in patients suffering from schizophrenia (39, 40). These results should be interpreted with caution, however, as they involved a non-standardised mindfulness practice that lacked appropriate supervision and was practiced much more intensely than is recommended (one case study reported 18 h of daily practice). The studies examined in this paper point clearly towards good tolerance and adhesion to mindfulness programs for FEP patients. In agreement with the effects of mindfulness found in patients suffering from schizophrenia (22), mindfulness-based interventions in FEP patients reduce depression and anxiety affects.

### Effects on Positive Symptoms

Very little evidence is available for FEP patients or UHR individuals concerning the effect of mindfulness-based interventions on positive symptoms like delusional ideas, bizarre perceptions, and hallucinations. Van der Valk and colleagues (36) reported no significant increase in positive symptom scores on the PANSS in FEP patients but Wang and colleagues (31) found a significant decrease in positive symptoms in the group that received a combined mindfulness + psychoeducation intervention. A similar result was reported by Usher and colleagues (33) for an intervention that did not include psychoeducation. It is clear that more data are needed in order to draw firm conclusions. In FEP patients and UHR individuals the practice of a mindfulness-based intervention can be considered as an activity that facilitates self-kindness and acceptance of emotional experiences, and that decreases stress associated with symptoms and even the intensity of the symptoms themselves. This would occur in a manner similar to that which takes place in mindfulness practice in depression prevention, in which the aim is not to prevent the occurrence of sadness and anxiety, but rather to break the vicious cycle that leads to relapse. This idea is supported by the results of a study by Dudley and colleagues (41) who performed a transversal study of 128 people with auditory verbal hallucinations and found a negative correlation between self-kindness skills and both distress levels associated with the voices and global severity level of the voices.

### Effect on Quality of Life and Functioning

The majority of studies discussed here report improved quality of life after a mindfulness-based intervention (22, 31–34, 37). Interestingly, improved quality of life is reported in both the quantitative (**Table 1**) and qualitative studies using a range of different psychometric scales. Improved self-esteem and self-efficacy was also reported in the MacDougall et al. (32) and Alvarez-Jimenez et al. (34) studies, in both FEP patients and UHR individuals. The aim of mindfulness practice is to help the person avoid engaging in automatic behavioural reactions and becoming overwhelmed by anxiety-provoking ruminations and negative thoughts (18, 19). The theory behind mindfulness groups is based upon such action mechanisms, and their clinical (28) and biological (42) effects have been observed in patients with major depression. A similar mechanism could underlie the effects in FEP patients and UHR individuals who practice mindfulness. Given the importance of improving quality of life (43), mindfulness practice could be of significant clinical interest in these populations. Despite the importance of emotional regulation for functioning, this was poorly assessed in the studies included in this review. Emotional regulation is a key skill trained in mindfulness-based programs and plays a critical role in relapse prevention of major depressive disorder (44). Thus, it would be of interest to compare emotional regulation in FEP patients and UHR individuals before and after their participation in a mindfulness-based program.

### Effect of Mindfulness on UHR Individuals

The study developed by Alvarez-Jimenez and colleagues (34) reported that a mindfulness-based intervention was of therapeutic interest for UHR individuals. In addition to acquiring mindfulness meditation skills, participants also improved their social functioning, personal skills, and self-esteem. Of the nine studies included in the present review, however, this study (34) was the only one that examined the effect of mindfulness in UHR individuals, which seriously limits generalization of their results to the population as a whole. The small number of studies with UHR individuals is likely due to the fact that these individuals seek care less often then FEP patients. This is due to the less pronounced severity of their symptoms, but also the difficulty of identifying this clinical profile. Despite the paucity of data, several arguments suggest that the practice of mindfulness could be beneficial for UHR individuals. First, paranoid symptoms are associated with a high level of self-criticism and low self-esteem (45, 46), and paranoid ideation and subclinical positive symptoms are associated with a low level of emotional regulation and a high level of impulsivity (47). These clinical features are regularly present in UHR individuals. Second, Collip and colleagues (48) have shown that mindfulness interventions can reduce low-intensity psychotic symptoms. In a randomized controlled trial of 130 patients with a history of depression and residual emotional dysregulation, they evaluated the effect of an MBCT intervention on intermittent paranoid symptoms and found a significant reduction in paranoid symptoms in the MBCT group in contrast to an increase in the control group. The authors suggested that MBCT could act upon psychotic symptoms when they are part of a continuum between the absence of positive symptoms and a confirmed psychotic spectrum disorder. That is, in clinical situations similar to those observed in UHR individuals. Further support for this idea comes from a study in healthy subjects in which it was shown that induced paranoid ideas were lessened by a mindfulness exercise, whereas they were more intense following a self-centered rumination exercise (49,).

### Methodological Limits

The results of this systematic review have a number of limitations. First, the small number of studies and their highly variable sample sizes (from 10 subjects in 29 to 128 in 31). Second, several of the articles included were open-labelled studies without a control group, and the main objective of the majority of studies was to evaluate feasibility. Of the nine studies reviewed only three had a control comparison group and two were randomized controlled studies. Third, only one study examining a mindfulness-based intervention in UHR individuals was included. It is important to note, however, that this should change in the near future, as there are a substantial number of ongoing studies that include this population. Finally, the various studies are difficult to compare because of their methodological heterogeneity. Indeed, a variety of mindfulness-based intervention protocols were used in the studies and only the studies developed by Tong and colleagues (37) and Wang and colleagues (31) used standardized protocols (MBSR and MBCT, respectively).

### Perspectives

As discussed above, investigations into the practice of mindfulness in FEP patients and UHR individuals are still in their infancy. Here, we propose several recommendations for future studies on mindfulness-based interventions in FEP patients and UHR individuals: i) include larger sample sizes in order to have more statistical power; ii) standardize the mindfulness protocol (program, number of sessions, length of sessions etc) in order to directly compare results from different studies; iii) include an active control group (relaxation, support group); iv) optimize and standardize the psychometric evaluation (CAARMS, PANSS, mindfulness scales; trait or state); v) avoid the inclusion of FEP patients only, and ideally compare effects in FEP patients versus UHR individuals; vi) provide long-term follow-up data after the intervention and collect key clinical elements like number of hospitalizations, suicide attempts, functional and well-being outcomes such as quality of life, daily life activities, school drop-out, and employment rates; vii) evaluate effects in UHR individuals in terms of psychotic transition; viii) conduct studies that include neuroimaging and biological data, in order to understand the underlying mechanisms of mindfulness-based interventions in FEP patients and UHR individuals. In light of these recommendations, a future unpublished study (the HORYZONS trial) aiming to include 170 FEP patients promises to be highly informative (51).

It is important to remember that cognitive problems, albeit less intense than in chronic schizophrenia patients, are present in FEP patients and UHR individuals (52, 53). Those functions most affected are working memory, attention, and social cognition. Even though mindfulness-based interventions are quite brief and highly structured, cognitive impairments can limit the access of these populations to MBCT or MBSR programs in an unchanged form. Considering the written guidelines for the use of mindfulness-based interventions in patients with chronic schizophrenia (38), we propose the following adaptations for mindfulness-based interventions in UHR individuals and FEP patients: i) maximal session length 1h30 and maximal duration of formal mindfulness exercises 20 min; ii) detailed debriefing of the metaphoric texts used in the mindfulness-based intervention; iii) avoidance of the use of the so-called noble silence for long periods.

## Conclusion

The practice of mindfulness has been shown to be efficient and feasible in patients with schizophrenia. Very few studies exist evaluating mindfulness in FEP patients and UHR individuals. Those that do exist show that the technique is well tolerated by FEP patients and affects symptoms of sadness and anxiety and perhaps some positive symptoms. Despite the small number of studies examining the neurobiological effects of mindfulness in FEP patients (and their complete absence in UHR individuals), these can easily be included in routine clinical practice making this a feasible, low cost, and promising avenue of future investigation. Even though these initial results are very promising, larger, more robust studies are necessary to draw any firm conclusions.

## Author Contributions

PV, FH, and JB contributed conception and design of the study. PV and CD organized the data search. PV and KR wrote the first draft of the manuscript. All authors contributed to manuscript revision, read and approved the submitted version. 

## Conflict of Interest

The authors declare that the research was conducted in the absence of any commercial or financial relationships that could be construed as a potential conflict of interest.
